# AMBIGUOUS LOSS AND RESILIENCE IN THE FAMILY CONTEXT OF DEMENTIA: A META-SYNTHESIS

**DOI:** 10.1093/geroni/igae098.2264

**Published:** 2024-12-31

**Authors:** Athena Chung Yin Chan, Cade Perkins, Lizbeth Garcia, Sophia Lyons, Frank Hernandez, Jonathan Singer

**Affiliations:** Texas Tech University, Lubbock, Texas, United States; Texas Tech University, Lubbock, Texas, United States; Texas Tech University, Lubbock, Texas, United States; Texas Tech University, Lubbock, Texas, United States; Texas Tech University, Lubbock, Texas, United States; Texas Tech University, Lubbock, Texas, United States

## Abstract

Having family members living with Alzheimer’s disease or related dementias (ADRDs) triggers high distress due to ambiguous loss. Ambiguous loss is an unclear loss with no resolution or closure to the physical presence and psychological absence of a loved family member. Resilience may buffer the distress from ambiguous loss in the family context of ADRDs. Existing reviews primarily focus on carer resilience. Little is known about the family processes navigating losses related to ADRDs. This meta-synthesis of qualitative studies aims to synthesize the family processes of ambiguous losses and resilience in the context of people with ADRDs. Peer-reviewed articles published after 1990 were identified in four electronic databases. A keyword search was performed associated with qualitative or mixed methods, dementia, and resilience or ambiguous loss. Seventy-two articles from 17 countries were included. Findings suggested the coexistence of resilience and ambiguous loss among persons with ADRDs and their family members. For individual-level and family-level resilience, three themes emerged, including belief systems (i.e., acceptance, gratitude, focusing on present and seeing positives in situations), organizational processes (i.e., finding support in friends and family, self-care and self-love), and communication / problem-solving (i.e., use of humor, working as a team). For intrapersonal and interpersonal ambiguous loss, two themes emerged including physical (i.e., loss of self-care, privacy and autonomy, current and expected future roles) and emotional (i.e., loss of identity, pride or dignity, sense of purpose, shame associated with memory loss, and home is no longer home). This meta-analysis calls for culturally-tailored family-based practice and policy.

